# Ranking Reputation and Quality in Online Rating Systems

**DOI:** 10.1371/journal.pone.0097146

**Published:** 2014-05-12

**Authors:** Hao Liao, An Zeng, Rui Xiao, Zhuo-Ming Ren, Duan-Bing Chen, Yi-Cheng Zhang

**Affiliations:** 1 Department of Physics, University of Fribourg, Fribourg, Switzerland; 2 Research Center of Complex Systems Science, University of Shanghai for Science and Technology, Shanghai, China; 3 Web Sciences Center, University of Electronic Science and Technology of China, Chengdu, China; University of Namur, Belgium

## Abstract

How to design an accurate and robust ranking algorithm is a fundamental problem with wide applications in many real systems. It is especially significant in online rating systems due to the existence of some spammers. In the literature, many well-performed iterative ranking methods have been proposed. These methods can effectively recognize the unreliable users and reduce their weight in judging the quality of objects, and finally lead to a more accurate evaluation of the online products. In this paper, we design an iterative ranking method with high performance in both accuracy and robustness. More specifically, a reputation redistribution process is introduced to enhance the influence of highly reputed users and two penalty factors enable the algorithm resistance to malicious behaviors. Validation of our method is performed in both artificial and real user-object bipartite networks.

## Introduction

With the rapid development of World Wide Web, our lives nowadays rely more and more on the Internet [1]–[4]. Online systems allow a large number of users to interact with each other and provide thousands of movies, millions of books, billions of web pages for them to choose [5]. Though a lot of useful online objects are out there, accurately ranking their quality is not easy. Therefore, many online websites (such as Ebay, Amazon, Netflix) introduce the so-called rating system [6], [7] in which users can evaluate objects by giving discrete ratings. To approximately judge the quality of a certain object, a user can refer to the historical ratings the object received.

The most straightforward method to rank objects is to consider their average ratings (we refer it as the *mean* method). However, such methods are very sensitive to the noisy information and manipulation. In these rating systems, some users may give unreasonable ratings because they are not serious about the rating or simply not familiar with the related field [8]. In addition, the system may contain some malicious spammers who always deliberately give high ratings to some low quality objects [9], [10]. To solve this problem, some ranking algorithms robust to spamming are proposed. Normally, these algorithms build a reputation system [11]–[14] for users. The ratings of users with higher reputation are assigned with more weight. By iteratively updating users' reputation [15], [16], the quality of objects can be ranked more accurately than the average ratings method. In fact, similar iterative ranking algorithms have been used in many other fields, such as country-product [17] or author-paper [18] systems.

Under this framework, some methods have already been proposed. A representative one is called iterative refinement (*IR*) method [19]. In IR, a user's reputation is inversely proportional to the difference between his or her rating vector and objects' estimated quality vector (i.e., weighted average rating). The estimated quality of objects and reputation of users are iteratively updated until they become stable. In [20], the iterative refinement algorithm is modified by assigning trust to each individual rating. More recently, another improved iterative method is designed (we refer it as the *CR* method) [21]. A user's reputation is calculated by the Pearson correlation [22], [24] between his ratings and objects' estimated quality. This method is claimed to be very robust to different spamming behaviors [25]–[27].

In this paper, we introduce a reputation redistribution process to the iterative ranking algorithm in [21], which can effectively enhance the weight of the highly reputed users and lower the weight of the users with low reputation in estimating the quality of objects. We test our method in both artificial and real data. The results show that the accuracy of objects' quality ranking is considerably improved. Moreover, we introduce two penalty factors to the iterative ranking algorithm which significantly improve its robustness against the malicious spamming behaviors. Interestingly, the improvement from the penalty factors is surprisingly large in real data, which indicates that there are many intentional pushing rating from spammers in real systems.

## Methods

### Iterative ranking algorithm with reputation redistribution

We first briefly describe the iterative algorithm with reputation redistribution (short for IARR). It is built directly on the CR method but with the reputation redistribution process for eliminating noisy information in the iterations, so as to improve the accuracy in objects' quality ranking. The rating system can be naturally described by a weighted bipartite network [28]. The users are denoted by set 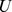 and objects (e.g. books, movies or others) are denoted by set 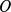. To better distinguish different type of nodes in the bipartite network, we use Latin letters for users and Greek letters for objects. The rating given by a user 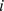 to object 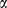 is the weight of the link, denoted by 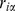. The degree of users and objects are respectively 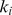 and 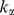. Moreover, we define the set of objects selected by user 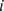 as 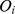 and the set of users selecting object 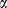 as 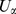.

We use 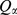 and 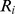 to note the quality of object 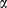 and the reputation of user 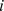, respectively. The initial configuration for each user is set as 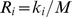 (where 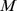 is the number of objects). The quality of an object depends on users' rating and can be calculated by the weighted average of rating to this object. Mathematically, it reads
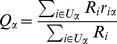
(1)


In the iteration, both 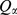 and 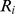 will be updated. To calculate the reputation 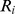 of user 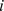 in certain step, we first calculate the Pearson correlation coefficient between the rating vector of user 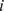 and the corresponding objects 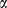 quality vector as the temporal reputation (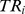):
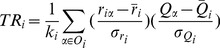
(2)where 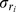 and 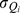 are, respectively, the standard deviations of the rating vector of user 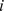 and the corresponding objects' quality vector, and 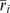 and 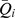 are their mean values. If 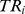 lower than 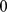, the reputation of user 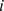 will be assigned to 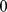. Therefore, 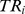 is bounded in [0,1]. As discussed in the introduction, the IR method considers a user's reputation as inversely proportional to the mean squared error between his/her rating vector and the corresponding objects' weighted average rating vector [19]. The reputation based Pearson correlation is shown to be more robust to spamming ratings than the IR method and thus lead to a more accurate estimation of object quality [21].


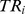
 is then nonlinearly redistributed to all users via
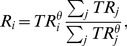
(3)where 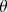 is a tunable parameter. The method will reduce to the mean and CR methods when 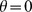 and 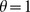, respectively [21]. The obtained 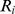 will be then used as the reputation of user 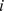 to calculate the quality of objects in eq. 1. With this reputation redistribution process, the user with high 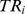 will be amplified, and vice versa. By reducing the weight of the users with low 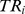, we can eliminate the noisy information in the iterative processes. This effect is accumulated in each iterative step, and will finally lead to a big improvement in the accuracy of object quality estimation. Actually, the basic idea of the reputation redistribution process is similar to the well-known *k-nearest neighbors* (KNN) algorithms which eliminate the noise by entirely drop the information of nodes outside the k-nearest neighbors [23]. The KNN algorithm is widely used in recommender systems. Here, we design a smooth way to implement the idea to object quality ranking. Though the modification of the method seems to be small, the improvement is substantial (see the following analysis).

Users' reputation and objects' quality will be updated in each step. The iteration stops when the change of the quality
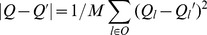
(4)is lower than a small value 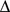 (in this paper, 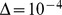).

### Improving the reliability of the method

We now try to enhanced the reliability of the method. In principle, when a user only assessed a small number of objects, he cannot have very high reputation. This is natural since it is easy for a user to guess correctly the quality of one object by chance, but very difficult for a large number. Therefore, when a user rates many objects and his reputation is still high, this user is more reliable. Similar idea is applied to the object side. If an object is rated by one or two users, though the ratings are high, it is too arbitrary to claim this object has high quality. Based on above two reasons, we introduce a penalty factor to eq. 1 and eq. 2, respectively. The modified eq. 1 reads
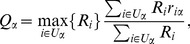
(5)and the eq. 2 is modified as



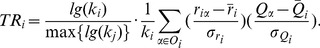
(6)With these two penalty factors, the objects rated by only low reputation users can only be low and the users who only rate a small number of objects cannot have high reputation. The penalty will be amplified in the iteration and finally filter out the influence of the not yet reliable users. This enhanced iterative algorithm is referred as IARR2 in the following text.

## Results on Artificial Networks

### Generating artificial networks

We start our analysis by applying IARR and IARR2 to artificial networks. To create the artificial network, we set 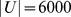,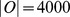. We assume that each object 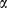 has an intrinsic quality denoted by 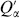. When a user 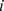 gives a rating to the object 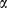, he/she will inevitably have some magnitude of rating error 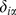. Accordingly, the rating to 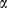 from user 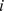 will be
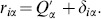
(7)


Without losing any generality, both users' ratings and objects' qualities are assumed to be 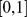. In our simulation, objects' qualities 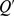 will be drawn from an uniform distribution 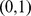. 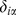 is draw from a normal distribution 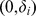 where 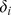 denotes the users magnitude of rating error. For each user 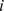, 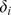 is generated from an uniform distribution 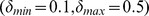.

To generate the user-object bipartite network, the rating (weighted links) will be added to the network one by one until the network reaches a certain sparsity (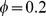). Under this setting, the final network will have 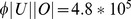 links. In most online systems, both users' and objects' degree follow quite broad degree distribution [29]. Accordingly, the preferential attachment mechanism is employed here to add links. At each step 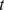, a random user 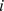 and a random object 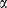 will be picked and a link will be added between them with the weight from eq. 7. The probabilities for selecting a user 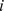 and object 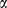 are respectively
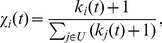
(8)and
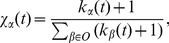
(9)where 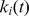 and 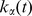 are the degree of user 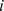 and object 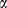 at time step 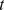 [30].

### Estimation of user reputation

For a good reputation estimation algorithm, the obtained user reputation 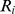 should be negatively correlated with 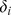. The stronger the correlation is, the better the algorithm is. Here, we compare the performance of IARR and IARR2 methods with the mean, IR [19] and CR [21] methods. The mean method is the most straight reputation estimation method in which user's reputation is calculated as one over the mean squared error between his/her rating vector and the corresponding objects' weighted average rating vector (without any iteration).

**Figure 1:**
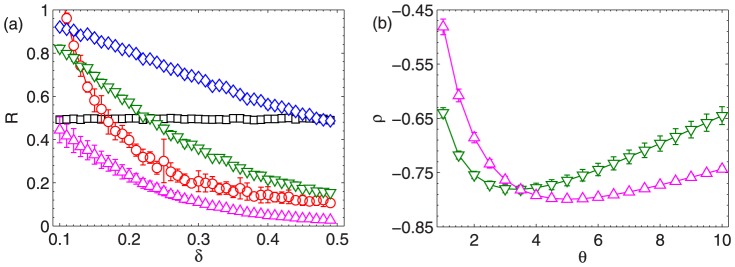
(a) The relation between 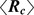 and 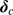 in different methods. The parameters are set as 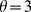 in IARR and 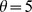 in IARR2. (b) the dependence of the Pearson correlation 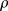 on 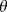 in IARR and IARR2 methods. The results in this figures are averaged over 10 independent realizations. The error bars are the corresponding standard deviations.

The results of each method are reported in Fig. 1. We define 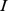 equally distributed intervals between 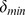 and 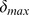 and group the nodes whose 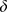 fall in the same interval. Each group is denoted by its median in 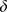 as 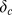. Since 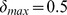 and 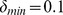, we set 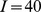 so that the interval is 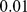. The averaged reputation 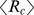 of the users in the same group is calculated. The relation between 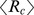 and 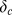 is reported in Fig. 1(a). Here, the parameter is set as 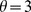 in IARR and 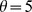 in IARR2. As one can see, 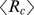 and 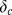 in most methods are negatively correlated except the mean method. In order to quantify the correlation, we calculate the Pearson correlation 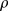 between 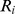 and 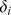. Specifically, 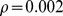 in the mean method, 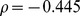 in the IR method, 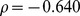 in the CR method, 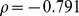 in IARR method and 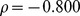 in IARR2 method. The dependence of the Pearson correlation 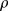 on 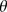 in IARR and IARR2 methods is studied in Fig. 1(b). Interestingly, there is an optimal 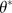 in both methods (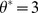 in IARR and 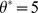 in IARR2). In the following analysis, we will set 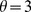 in IARR and 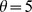 in IARR2.

### Robustness against random and malicious ratings

A good ranking algorithm should be not only accurate in estimating users' reputation and objects' quality, but also robust against distort information, i.e. the accuracy of the algorithm shouldn't be strongly affected when the system contains some random or malicious ratings. The random ratings mainly come from the naughty users who just play around with the information and give ratings which mean nothing. The malicious ratings are from some spammers who always gives maximum/minimum allowable ratings that also try to push up some target objects. Both type of distort ratings widely exist in real systems [31], [32]. Therefore, we investigate the effect of the noisy and willful distort ratings on the performance of the IARR and IARR2 methods.

We start with the system with random ratings. We first generate the artificial networks according to the rules described above. In order to add some noisy information to the systems, we randomly pick 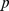 fraction of the links and replace the rating on each of these links by a random value in range of [0,1]. Clearly, the noisy information in the system gradually increases with the parameter 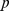. When 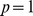, there is no any true information in the rating system. In the following analysis, we set 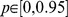.

In order to compare the performance of different ranking algorithms, we here adopt two metrics: Kendall's tau [33] and AUC (the area under the receiver operating characteristic curve) [34]. The Kendall's tau here measures the rank correlation between the estimated quality of objects 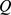 and the “true”quality of them 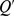. Mathematically, it reads
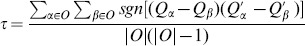
(10)where 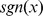 is the sign function, which returns 1 if 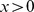; −1 if 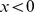; and 0 for 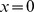. Here 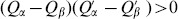 means concordant and negative means discordant. According to the definition, 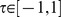. A higher 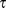 indicates a more accurate estimation of objects' true quality.

In real cases, the true quality of objects is unknown, which makes it impossible to evaluate the algorithm by 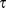. Therefore, we consider another accuracy measure called AUC. To calculate AUC, one should select a group of benchmark objects which are considered to be generally with high quality. We selected 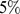 objects with highest 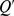 as the benchmark objects. The AUC requires 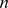 times of independent comparison of the benchmark objects and non-benchmark objects. After the comparison, we record 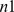 as the number of times in which the benchmark object has higher 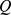 than non-benchmark object, and 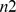 as the number of times in which the benchmark object and the non-benchmark object are having the same 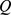. The final AUC is calculated as 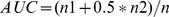. If all the objects are ranked randomly by some algorithm, 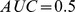. When 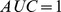, all the benchmark objects are ranked higher than the non-benchmark objects.

**Figure 2:**
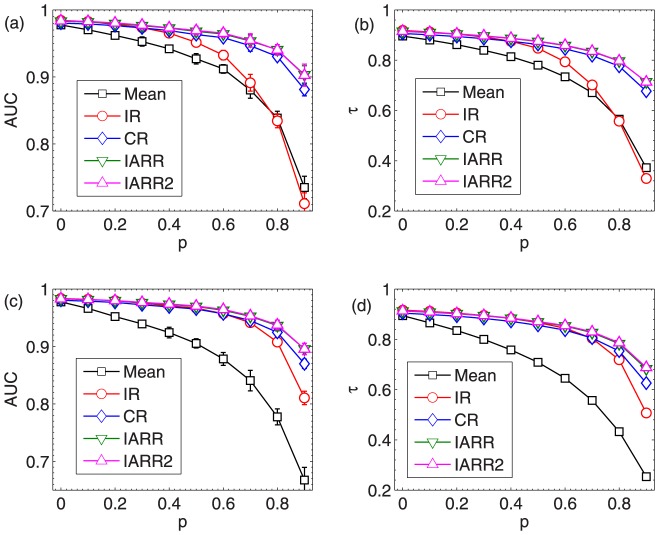
(a) and (b) the AUC and 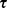 of different algorithms to random rating spamming. (c) and (d) different algorithms to malicious push rating spamming. The results in this figure are averaged over 10 independent realizations. The error bars are the corresponding standard deviations.

Here, we compare the Kendall's tau and AUC in five algorithms: Mean, IR, CR, IARR and IARR2. In Fig. 2(a) and (b), we respectively report the dependence of 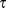 and AUC on 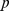 in different algorithms. As one can see, IARR and IARR2 methods outperform the other three methods, especially when 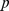 is large. However, the difference between IARR and IARR2 algorithms is almost indistinguishable. This is due to the reason that the random rating attack cannot fully model the spamming behavior in real systems.

We further consider the malicious rating attack in the artificial networks. In practice, we randomly pick 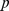 fraction of the links in the generated artificial network and set half of them to be the maximum rating (i.e. 1) and the other half of them to be the minimum rating (i.e. 0). This scenario models the so-called push rating in which spammers try to promote the target low quality objects. The results of 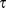 and AUC of different ranking algorithms in this case are shown in Fig. 2(c) and (d). One can observe that IARR and IARR2 still have advantage over other methods.

**Figure 3:**
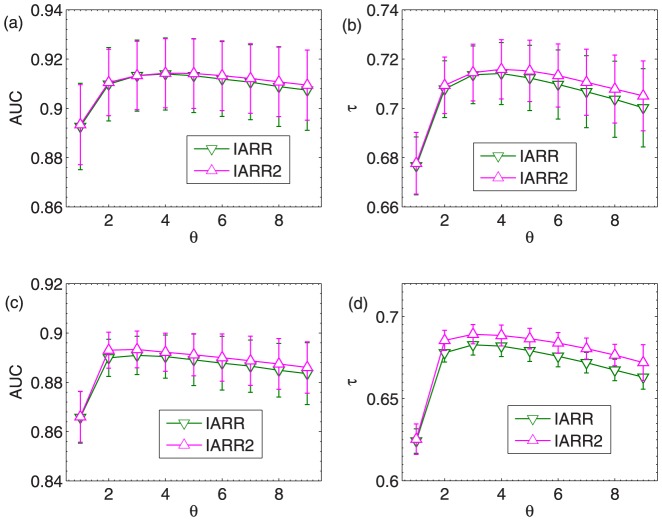
(a) and (b) the dependence of AUC and 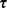 on 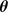 in IARR and IARR2 methods in the random rating attack case. (c) and (d) the dependence of AUC and 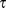 on 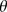 in IARR and IARR2 methods in the malicious rating attack case. The results in this figure are averaged over 10 independent realizations. The error bars are the corresponding standard deviations.

The parameters are respectively set as 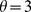 and 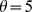 in IARR and IARR2 in the robustness analysis above. In Fig. 3, we analyze the effect of 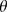 on the resultant AUC and 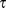 in these two methods. We set 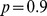 in both random rating and malicious rating attacks. The results show that the parameter 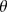 can indeed improve the performance of the ranking algorithms (Note that when 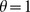, IARR degenerates to the CR algorithm). Moreover, we can observe that the optimal 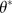 in IARR and IARR2 are more or less the same. Specifically, 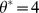 in the random rating attack case, and 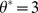 in the malicious rating attack case. Finally, it shows that IARR2 enjoys a higher AUC and 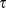 than IARR in the malicious attack case, which implies that IARR2 may have high performance in real systems (since the malicious ratings are more common in real case).

## Results on Real Networks

In this section, we will study the IARR and IARR2 methods in real systems. Here, we select two commonly used real data sets containing ratings on movies: Netflix and MovieLens. MovieLens is provided by GroupLens project at University of Minnesota (www.grouplens.org). We use a subset of the complete data. In our subset, there are 1 million ratings given on the integer rating scale from 1 to 5. Each user in subset has at least 20 ratings. Netflix is a huge data set released by the DVD rental company Netflix for its Netflix Prize (www.netflixprize.com). We again extracted a smaller data set by choosing 5000 users who have rated at least 20 movies (the same as MovieLens) and took all movies they had rated. The Netflix ratings are also given on the integer rating scale from 1 to 5. Some basic characteristics of these data sets are summarized in table 1.

**Table 1:** Some basic characteristics of the real data sets considered in this paper.

Methods	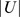	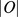	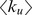	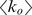	Sparsity
MovieLens	6040	3706	166	270	0.0447
Netflix	5000	16195	214	66	0.0132

**Figure 4:**
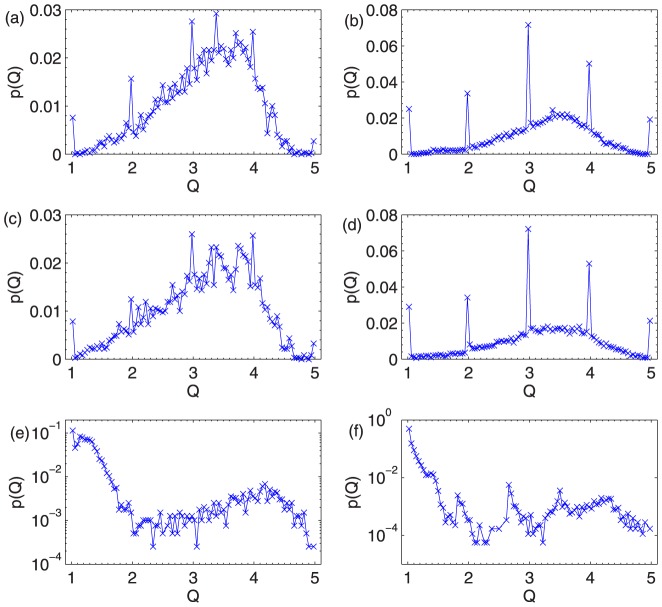
(a), (c) and (e) are the distribution of 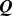 of the CR, IARR and IARR2 algorithms in Movielens data, respectively. (b), (d), (f) are the distribution of 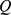 of the CR, IARR and IARR2 algorithms in Netflix data, respectively. 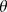 is set as 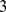 in both IARR and IARR2 algorithms.

**Figure 5:**
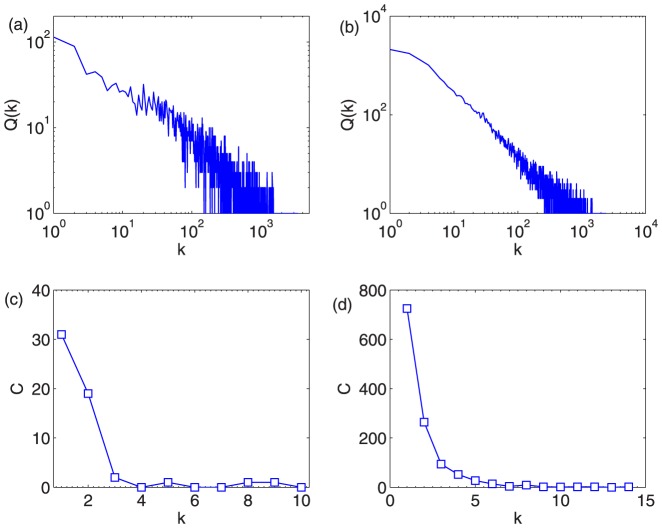
(a) and (b) are the frequency distribution of object degree in Movielens and Netflix, respectively. (c) and (d) are the relation between frequency 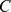 and 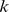 in movielens and Netflix, respectively.

We run different ranking algorithms in these two data sets and study the distribution of the obtained 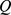. As shown in Fig. 4, 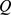 of both CR and IARR algorithms roughly follow a normal distribution. One can also see that there is an abrupt peak in each integer rating, especially in the Netflix data. This is because some objects are only rated by one user, or all users give the object with the same rating. We further study the occurring frequency of this case in real online systems. We first study the degree distribution of objects in the real systems. Fig. 5(a) and (b) show the frequency distribution of object degree in Movielens and Netflix, respectively. One can clearly see that both distributions follow power-law form. Another message one can get from these two figures is that there are many objects are only rated by one user, around 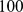 objects in Movielens and 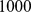 in Netflix. Once these objects are rated with 5, they will be considered as the highest quality objects by the mean and CR method. Furthermore, we check the frequency of these low degree objects with high ratings. Here, we select the object with the same degree 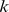 and calculate the frequency 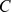 that all raters give them high ratings (in our case, we consider rating 4 and 5 as high ratings). In Fig. 5 (c) and (d), we show the relation between frequency 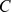 and 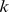 in movielens and Netflix, respectively. As one can see, the value of 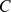 is rather big, especially when 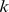 is small. These objects, though with low degree, will be considered as highest quality objects by the mean and CR method.

The above analysis implies that the ranking provided by CR and IARR algorithms are not very reliable since many small degree objects will appear in the top of quality ranking list. This problem is well solved in the IARR2 method. With the penalty factors, IARR2 will give low score to those suspicious objects (i.e. objects with high rating but small degree). In Fig. 4, we can see that the abrupt peak disappear in the 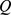 distribution from the IARR2 algorithm. The penalty factors will decrease the maximum value of 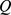. For better illustration, the distribution of 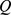 in the IARR2 is rescaled to [1], [5] in Fig. 4. We remark that the object ranking from the IARR2 algorithm can well reflect objects' true quality. We will use some awarded movies to support this statement in the following.

Since we don't know the true quality of the movies in these two data sets, we adopt the AUC metric to study the IARR and IARR2 here. To calculate the AUC, we select those movies which nominated at Annual Academy Award (source:www.filmsite.org) as benchmark good movies. In movieLens and Netflix data contains 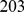 benchmark movies and 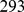 benchmark movies.

Table 2 shows the AUC resulted from four different algorithms applying to the real data sets. One can immediately see that the AUC is generally lower in the Netflix data, which indicates that there are more spammers (or more harmful spammers) in Netflix data. Moreover, it shows that the CR method doesn't actually have significant advantage towards the Mean and IR methods, though it largely outperforms the Mean and IR methods in the artificial networks. This result indicates that the CR method is very sensitive to the “real”spammers. The IARR can slightly improve the performance of CR method by introducing the reputation redistribution process (the parameter is set as 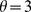 here). Interestingly, the IARR2 method remarkably outperform all the other methods. This implies that the IARR2 method indeed captures the harmful features of the real spammers. More specifically, the IARR2 method is very robust against the cases where low quality objects are highly rated by several unreliable users. Moreover, it also punishes some spamming users who want to increase their reputation by giving several movies the mean ratings. The results in table 2 indicates that these spamming behaviors happen frequently in real online rating systems.

**Table 2:** AUC values of different algorithms for the real-data sets.

Methods	Mean	IR	CR	IARR	IARR2
MovieLens	0.873	0.876	0.872	0.876	**0.902**
Netflix	0.729	0.746	0.746	0.758	**0.886**

## Conclusions

In this paper, we propose a robust iterative ranking algorithm with reputation redistribution process. The reputation redistribution process can effectively enhance the weight of the highly reputed users and reduce the weight of the users with low reputation in estimating the quality of objects. Two penalty terms to the iterative ranking algorithm which significantly improve its robustness against some malicious spamming behavior. We test our method in both artificial and real data. The results show that the accuracy of ranking the quality of objects is considerably improved. Interestingly, the improvement from the penalty terms is surprisingly large in real data, which implys that there are many intentional pushing rating from spammers in real cases.

Finally, we remark that our work is of great significance from practical point of view. Nowadays, the internet plays a significantly important role in our daily lives. Online users usually select products by referring to peers' ratings. Without a reputation system, there is a risk that users' choices might be misled by some spamming ratings. Our method in this paper is not only effective in estimating the true quality of the objects but also very robust to spamming ratings. Therefore, we believe that our method can be very useful when applied to real online websites.
